# Historical Westward Migration Phases of *Ovis aries* Inferred from the Population Structure and the Phylogeography of Occidental Mediterranean Native Sheep Breeds

**DOI:** 10.3390/genes13081421

**Published:** 2022-08-10

**Authors:** Yousra Ben Sassi-Zaidy, Aziza Mohamed-Brahmi, Melek Chaouch, Fabio Maretto, Filippo Cendron, Faouzia Charfi-Cheikhrouha, Souha Ben Abderrazak, Mnaour Djemali, Martino Cassandro

**Affiliations:** 1 Laboratory of Diversity, Management and Conservation of Biological Systems, LR18ES06, Faculty of Sciences of Tunis, University of Tunis El Manar, Tunis 2092, Tunisia; 2 Department of Agronomy, Animal, Food, Natural Resources and Environment, University of Padova, 35020 Legnaro Padova, Italy; 3 Laboratory of Animal Genetic and Feed Resources Research, Department of Animal Science, Institut National Agronomique de Tunis (INAT), University of Carthage, Tunis-Mahragène Tunis 2078, Tunisia; 4 Laboratory of Agricultural Production Systems Sustainability in the North Western Region of Tunisia, Department of Animal Production, Ecole Supérieure d’Agriculture du Kef Boulifa, University of Jendouba, Le Kef 7119, Tunisia; 5 Laboratory of Medical Parasitology, Biotechnology and Biomolecules (LR11IPT06), Institut Pasteur de Tunis, Tunis 1002, Tunisia; 6 Laboratory of Bioinformatics, Biomathematics and Biostatistics (LR16IPT09), Institut Pasteur de Tunis, Tunis 1002, Tunisia

**Keywords:** Mediterranean native sheep, phylogeography, population structure, microsatellite markers

## Abstract

In this study, the genetic relationship and the population structure of western Mediterranean basin native sheep breeds are investigated, analyzing Maghrebian, Central Italian, and Venetian sheep with a highly informative microsatellite markers panel. The phylogeographical analysis, between breeds’ differentiation level (Wright’s fixation index), gene flow, ancestral relatedness measured by molecular coancestry, genetic distances, divergence times estimates and structure analyses, were revealed based on the assessment of 975 genotyped animals. The results unveiled the past introduction and migration history of sheep in the occidental Mediterranean basin since the early Neolithic. Our findings provided a scenario of three westward sheep migration phases fitting properly to the westward Neolithic expansion argued by zooarcheological, historical and human genetic studies.

## 1. Introduction

The Neolithic Revolution is considered a pivotal event in modern human history [1]. Coined as the transition from a “hunting-gathering” to “farming-herding” life style, the Neolithization was started in the Fertile Crescent somewhere in South-West Asia around 12,000 years ago [2,3,4]. Cultivating plants first and, a little later, domesticating animals, is considered the center of the Neolithic Revolution [5]. Sheep, contemporaneously with goats, were the first livestock species domesticated as the food items in the Fertile Crescent in the Holocene some 12,000 to 10,000 years ago [2,6,7]. It was approved recently that domestic sheep *Ovis aries* (*O. a.*)were domesticated from a unique wild species, the Asiatic mouflon *Ovis orientalis gmelinii* named *Ovis gmelinii* (*O. g.*), in the region between Northern Zagros to Southeastern Anatolia [8,9]. From this Neolithic center of domestication, and due to the increasing population size of the first villages, Neolithic farmers with their Neolithic tools, plants and animals began to spread beyond Southwest Asia into Europe and North Africa, making a agriculture dispersal westward [5]. In fact, livestock species, including sheep, followed human Neolithic migrations westward, colonizing Cyprus between 10,500 to 9000 Y.B.P. and reaching South Europe by approximately 8500, as well as the Maghreb (Northwest Africa), probably by 7500 Y.B.P. [10,11]. The Mediterranean basin is considered as a main thoroughfare for the maritime diffusion of small ruminant species into South Europe and North Africa [5,7,10]. Besides this maritime way, two terrestrial currents were also defined as a way of migration of Neolithic Middle Eastern farmers and their livestock: (i) the Danubian current, which allowed the arrival of breeding in Northern Europe [12,13,14,15,16] and (ii) the Suez Isthmus and/or the South of the Sinai Peninsula allowing the arrival by transhumance in North Africa [5,8,17].

Studying the genetic structure of the early domesticated animal species by the first Neolithic human populations gives valuable insight into the history of modern human populations and their migration routes [7]. Many studies have provided useful insights into the movement of sheep pastoralism from the domestication center to Eurasia [15,16,18,19,20,21]. In addition, the gene flow between American and Near Eastern sheep breeds was explored by Blackburn et al. [22]. The usefulness of microsatellite markers in the estimation of sheep populations’ genetic structure and breeds’ divergence was highlighted by several studies on both Mediterranean coasts [23,24,25,26,27,28,29,30,31]. Despite the important role of the Mediterranean basin in the diffusion of sheep to the European and African coast since the beginning of the Neolithic, no studies have been focused on the assessment of the westward Mediterranean sheep migration, neither by sea, nor by land routes, including simultaneously Northern and Southern occidental Mediterranean sheep breeds, except for the recent investigations of sheep breeds from the Maghreb, Tunisian, Algerian and Moroccan [32,33,34] that have reported the origin and the migration events of the analyzed breeds.

The aim of our study is to explore the expansion of *O. a.* across the Mediterranean basin through analyzing native sheep breeds originating from the North and South coasts of the Western Mediterranean Sea, on the basis of neutral microsatellite markers. The specific objectives of this work are to disclose the colonization history and the divergence of the Near Eastern ancestor domestic sheep following both North and South lanes of the Mediterranean coasts, by analyzing very old and native sheep breeds from Italy and Maghreb region (Tunisia, Algeria and Morocco) using a panel of seventeen informative microsatellites markers.

## 2. Materials and Methods

### 2.1. Sampling Design and Genotyping

#### Population Samples

Blood samples were collected from 975 sheep listed in 11 Mediterranean breeds and divided into 2 groups: Maghrebian (Southwestern Mediterranean coasts) and South European (Northwestern Mediterranean coasts). The Maghrebian group enclosed (1) three old and native and one crossbred sheep breeds: the oldest and specific fat-tailed sheep of Tunisia (Barbarine, BAR, *n* = 64); the Ouled Djellal breed, known as the oldest and specific sheep of Algeria, which was introduced in Tunisia in the last century and renamed as the Western Thin Tailed breed (QFO, *n* = 41) due to its thin tail and in relation to its Western origin related to the Tunisian–Algerian border; the crossbred (CRO, *n* = 30) forming the first generations of BAR × QFO crossing; and a very old breed of Maghrebian Sahara introduced in Tunisia from Moroccan oasis in the few last decades (D’man, DM, *n* = 28); (2) two modern sheep breeds created in Tunisia in the last century by crossing Mediterranean breeds: the Sicilo–Sarde breed (SS, *n* = 45) resulting from a crossbreeding between two islander Italian breeds (Comisana from Sicilia and Sarda from Sardinia); and the “Noire de Thibar” breed (NTH, *n* = 41) created by crossing the French Merino d’Arles with the Algerian Ouled Djellal.

The South European group enclosed samples collected from the ancient Italian native breeds belonging to 2 locations: (1) Northeastern Italy for the Venetian native breeds Alpagota (ALP, *n* = 250), Brogna (BRO, *n* = 186), Foza (FOZ, *n* = 118) and Lamon (LAM, *n* = 141); and (2) Central Italy for the Appenninica (APP, *n* = 31) native breed. Sheep individuals of both groups were sampled by official veterinarians of the local health authorities during sanitary control of the herds; thus, ethical review and approval were waived for this study and no specific authorization from an animal ethics committee was required. Details about the morphological characteristics of the Maghrebian [35,36] and the Italian [37] sampled breeds are summarized in Table 1.

Genomic DNA extraction was carried out from 300 µL whole blood using the Wizard Genomic DNA Extraction Kit (Promega, Madison, WI, USA) following the manufacturer’s protocol. The entire number of samples was genotyped at 17 microsatellites loci panel (Table 2) established from the ISAG recommended microsatellites markers [38] and from previous sheep genetic diversity studies [39,40,41]. Genotypes for all 17 microsatellite markers were determined by means of three multiplex PCR reactions using fluorescence-labeled primers in a total volume of 12.5 µL. Amplification was performed using standard PCR reactions in a GeneAmp 9700 thermal cycler (Life Technologies, Carlsbad, CA, USA) starting from 50 ng of purified DNA. The 17 microsatellites were amplified with the following conditions: an initial denaturation step of 5 min at 95 °C, 35 cycles of 30 s at 95 °C, 1 min 30 s at 61 °C and 30 s at 72 °C and a final extension of 30 min at 60 °C. Multiplexes pooled by capillary electrophoresis and allele size were performed with a CEQ 8000 Genetic Analysis System (Beckman Coulter, Fullerton, CA, USA).

### 2.2. Statistical Analysis

Multilocus individual genotypes were checked for null alleles and errors using MICROCHECKER 2.2 [42] and MSA v.4.05 [43]. The full data set enclosing all individuals and all markers was used to perform descriptive statistical analysis of genetic diversity at microsatellites and breeds, as mentioned by [29]. The genetic relatedness between populations and phylogeographic analysis was performed in terms of: the rate of gene flow across the breeds (Nm), expressed as the number of migrants per generation [44], the Wright’s fixation index (F_ST_) [45], calculated using GENETIX version 4.05.2 [46], and the remote (ancestral) relatedness among populations estimated by measuring between-breed molecular coancestry (*f_ij_*) using Molkin 3.0 [47]. Furthermore, ARLEQUIN software [48] was used to perform the evolutionary relations of the analyzed populations by measuring both genetic distances: Nei’s standard distance (D_S_) [49], recommended for studying the fine-scale population differentiation [50,51], and (δµ)^2^ distance, appropriate for resolving the relationships among separate populations and estimating evolutionary times based on the microsatellites’ data [50,52,53]. Neighbor-joining (NJ) trees basing on Ds and (δµ)^2^ were constructed using the PHYLIP package [54]. Bootstraps of 1000 replicates were performed to test the robustness of the trees’ topology. The dendrogram was depicted using MEGA 5 [55]. To examine the genetic structure of the analyzed Mediterranean breeds and to assess the degree to which breeds differ from each other, two methods were completed using the Discriminant Analysis of Principal Components (DAPC) implemented in the adegenet package R [56] and the Bayesian clustering, implemented by the STRUCTURE version 2.3.4 software [57] based on the most likely number of clusters (K) in the dataset. For this last method, the optimum number of clusters fitted to the data was established following the ΔK method [58]. The output obtained was used directly as input data in the cluster visualization programs DISTRUCT [59] and CLUMPAK [60]. The GPT (Global Positioning Trees) web-server [61] was used to map the (δµ)^2^ phylogenetic tree on a virtual globe viewed on Google Earth in order to illustrate the Phylogeography of the analyzed breeds.

## 3. Results and Discussion

### 3.1. Microsatellites’ Performance

The results demonstrated no evidence for genotyping errors, as no presence of null alleles or disequilibrium linkage between loci were detected. Hence, all the 17 loci were considered in the dataset. A total number of 383 alleles were detected across the 17 microsatellites markers assessed in the 975 genotyped animals. The number of alleles ranged from 16 to 38 with a mean of 22.53 ± 6.07. The mean PIC is 0.80 ± 0.08 highlighting the usefulness of this highly polymorphic and informative panel of microsatellites (Table 2). The average F_ST_ value reaching 0.080 (*p* < 0.001) indicated a moderate differentiation level between the different Mediterranean breeds. In fact, only 8% of the total genetic variation was detected among breeds, and the main part (92%) of the variability was revealed among individuals. The usefulness of microsatellite markers in genetic diversity and divergence within and between sheep populations or breeds is proven [62]. These markers constitute the most frequently used marker for genotyping local farm animal breeds in the two last decades [63].

### 3.2. Mediterranean Sheep Breeds’ Divergence and Relationships

#### 3.2.1. F_ST_ Distances and Neighbor Network

The differentiation level between the analyzed occidental Mediterranean breeds was measured by the pairwise F_ST_ values (Table 3). F_ST_ values ranged within the same group (Maghrebian or South European) from 0.005 to 0.043, reflecting a low within the groups’ differentiation level, excepting a (1) ALP/FOZ differentiation slightly exceeding the low level (F_ST_ = 0.053), and (2) as unexpected, the APP/Venetian breeds differentiation level demonstrated the greatest level of divergence, which is higher than the DM/Venetian breeds’ differentiation. Until this present study, no available genetic data has highlighted this later relationship between the North-Saharan DM breed and the Venetian sheep breeds. The fact that an APP central Italian breed was shown to be more differentiated from North Italian Venetian breeds (F_ST_ ranging from 0.116 to 0.147) than from Maghrebian breeds (F_ST_ ranging from 0.052 to 0.073) and, even closer to QFO and SS (F_ST_ = 0.052 and 0.057, respectively). These F_ST_ values can be explained by an old gene flow insured by the active maritime route between the West-Centre of Italy (origin of APP), the two Italian islands, Sicilia and Sardinia (Origin of the SS ancestors, Comisana and Sarda respectively), and the North-East of Maghreb (origin of Ouled Djellal breed and direct ancestor of the Tunisian QFO). Gaouar et al. revealed a differentiation level, reaching 8% between some local Algerian breeds (Hamra, Sidauo, D’man) [25] and 6.1% between French and North African breeds [27], which are greater than the differentiation level between APP/QFO (5.2%). Trouette [64] has proposed a Roman origin of the Algerian Ouled Djellal with a hypothesis highlighted by Djamaï and Zebiri, [65] who emphasized the presence of a morphologically similar breed to the Algerian Ouled Djellal, which was, until presently, in the “Taranto” region in Italy. To assess the differentiation level among more geographically close regions, we further calculate the F_ST_, removing the Venetian breeds. The results indicated that the differentiation level among breeds dropped from 8% to 3% between APP and the Maghrebian breeds. This differentiation level (3%), which is higher than the one revealed within Tunisian breeds (2.1%, [24]), but lower than the differentiation within Moroccan (3.6%, [30]), Algerian (0.9% to 8.5%, [25]) and Ethiopian (5.3% [66]) sheep breeds, revealed the closeness of the Center Italian APP and the occidental Mediterranean basin North African native breeds. Accordingly, our results could confirm the hypothesis of an ancestral relationship that is more or less ancient (prehistoric or antiquity periods) between the North African woolly thin-tailed sheep group (Ouled Djellal-like sheep) and the Central Italian APP sheep belonging to the same group.

On a larger scale, this Mediterranean differentiation level is lower than the continental level of African sheep including North, East and South African sheep breeds (12.43%), which is revealed by Edea et al. [66]. Lawson Handley et al. [67], analyzing differentiation level of Southern and Northern European sheep breeds together with some near Eastern ones, revealed a higher differentiation level between the two Italian breeds, Comisana and Sarda (10.3%), than between the Sarda and Awassi native near Eastern fat-tailed breed (5%). These findings highlighted the ancient gene flow between Mediterranean breeds before their recent isolation.

**Figure 1:**
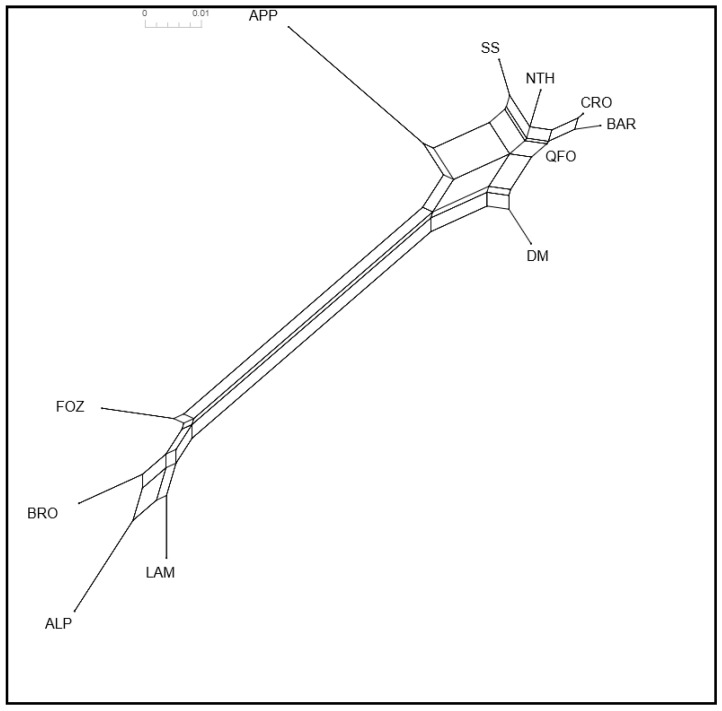
Neighbor network demonstrating the genetic relationship between the Mediterranean analyzed breeds. Venetian breeds: Alpagota (ALP), Brogna (BRO), Foza (FOZ) and Lamon (LAM); Central Italian breed: Appenninica (APP); Maghrebian breeds: Barbarine (BAR), Western thin-tailed breed (QFO), crossbred BAR × QFO (CRO), D’man (DM), Sicilo–Sarde (SS) and Noire de Thibar (NTH).

The differentiation relationships between the analyzed Mediterranean sheep was illustrated in the neighbor network built from the F_ST_ distances (Figure 1). The graph clearly separated the group of the Venetian breeds from the group of the North African-Central Italian sheep breeds. The Central Italian APP breed clustered into the same sub-branch of the South European’s origin of North African breeds (SS, from the Italian Comisana and Sarda, and NTH, from the French Merino d’Arles). The relationship between the DM and the Venetian breeds is illustrated by the long articulated sub-branch grouping them, indicating the very old relatedness between these two geographically separated groups.

#### 3.2.2. Molecular Co-Ancestry and Gene Flow

To identify the historical relationships between the analyzed Mediterranean breeds, the migration rates on different time scales, ancestral and more recent patterns of migrations were estimated, respectively, in terms of between breeds, molecular co-ancestry indexes (*f_ij_*) and effective migrant per generation (Nm) (Table 4).

Gene flow rates (Nm) among breeds, varying from 1.25 to 50.65, were considered moderate to high. The lowest number of migrants was noted within the Italian breeds, between the ALP Venetian breed and the central Italian APP. A low level of Nm (from 3.06 to 5.27) was detected within the Venetian breeds compared to the higher level of migration (from 5.60 to 50.65) within the Maghrebian group. These results indicated a high degree of differentiation between the Venetian breeds despite their belonging to a very narrow geographical region, interpreted as a “Wahlund” effect in this group. The highest values of gene flow (ranging from 30.54 to 50.65) reflected on the one hand, the actual gene exchange between BAR, QFO and their crossbred CRO recently practiced in Tunisia in the last few decades and, on the other hand, the old miscegenation between the Algerian’s originated thin-tailed sheep (QFO) and the BAR fat-tailed sheep, both considered as native Maghrebian breeds [29].

The considerable Nm value (18.13) revealed between NTH and QFO highlighted the contribution of the QFO gene pool during the NTH breed creation by crossing the Algerian Thin-tailed “Ouled Djellal” (from which QFO was derived from) with the French Merino d’Arles in 1924 [24]. The center Italian APP breed shared a higher migrant number with the Maghrebian breeds (3.20 < Nm < 4.55) than with the Venetian breeds (1.25 < Nm < 1.85). The highest gene flow (Nm = 4.55) between APP and Maghrebian breeds was revealed between APP and QFO, originating from the Algerian “Ouled Djellal” breed, highlighting a shared gene pool between these two Maghrebian and Italian sheep breeds. A similar gene flow level (Nm = 4.06) was revealed between the APP and SS breed descendants of the Sicilian and Sardinian island breeds, Comisana and Sarda, respectively. These results, in accordance with the F_ST_ distances ones, indicated that past sheep exchanges (since the Carthaginian and then the Roman times and/or more ancient, from the Neolithic) were practiced between the two close shores of the Mediterranean basin: the northern shore (central and southern Italy) and southern shore (Tunisia and Algeria), going through the Tyrrhenian island. This exchange, exceeding the one taking place between North Italian (Venetian) and central Italian breeds, incited us to highlight two different introduction waves/routes for Italian sheep, a northern introduction by land through the Danubian valley route and a southern maritime route reaching the central and southern Italian peninsula. As unexpected, a moderate number of migrants (Nm = 3.20) was detected between APP center Italian breed and the DM breed originating from the southern oasis of Morocco. This migration rate, higher than the migration level detected between the APP and Venetian breeds (varying from 1.25 to 1.85) revealed a very ancient relationship between the Maghrebian and Italian sheep, surely older than the known historical relationships starting at the Carthaginian and then Roman times and probably going back to the Neolithization of the occidental part of the Mediterranean coasts. Furthermore, the reported gene flow between the APP and Venetian breeds (ranging from 1.25 to 1.85) is lower than the gene flow between the DM and Venetian breeds (ranging from 1.74 to 2.03), which presumed a very ancient gene flow between the ancestral sheep that reached Europe by land and the ancestral sheep of North Africa, which would have introduced by land via Ishm Sues, and/or by sea. These two ancestors would have been derived from the ancestral sheep of the domestication center and would be included within the first waves of migrations towards the West. These purposes will be ascertained using the molecular ancestry correlation between these sheep groups from the Northern and the Southern coasts of the Mediterranean basin, measured using the *f_ij_* molecular co-ancestry coefficient. The ability of this coefficient to assess the genetic differentiation of an ancestral origin was proven by Alvarez et al. [51].

The paired molecular co-ancestry matrix is shown in Table 4, with values below the diagonal. It provided additional information, better clarifying the ancestral relationships before the breeds’ separation. The highest *f_ij_* values (ranging from 0.17 to 0.21) were revealed between Venetian breeds and were slightly higher than those recorded between the Maghrebian breeds (0.15 < *f_ij_* <0.19). The results that did not correlate with those of the Nm parameter highlighted the ancestral identity that existed between the Venetian breeds before their recent isolation and the strong discrimination of the recent known breeds, sharing the lowest values of the effective number of migrants (Nm) that reflected the recent relatedness more than the remote relative genetic contribution among breeds. The lower values of *f_ij_* between the Maghrebian breeds demonstrated that the identity between their founding populations was not as strong as between the ancestral populations of the Venetian breeds, which suggested more than one introduction wave. It is not less important here to demonstrate that the identity between the founding populations of the Maghrebian breeds and the APP (*f_ij_* varying from 0.15 to 0.17) is greater than that reported between the APP and the Venetian breeds (0.11 < *f_ij_* < 0.12). This finding further illustrated the very ancient relationship between the APP and Maghrebian indigenous breeds, DM, BAR and Ouled Djellal’s originated QFO, especially that neither the geographical nor historical link was revealed between the South Moroccan DM breed and APP (*f_ij_* = 0.15). The *f_ij_* values detected between the Maghrebian breeds and the Venetian breeds, varying from 0.09 to 0.12, highlighted the presence of a significant ancestral relationship between these two groups. In fact, the level of the ancient identity between the South Maghrebian DM breed and the Venetian breeds (*f_ij_* = 0.10 and 0.12) is closer to the one between the APP and Venetian group (*f_ij_* = 0.11 and 0.12). This ancestral relationship seems to be very ancient, going back to the first waves of westward sheep migration from the center of domestication, dating from the earliest Neolithic period of the occidental Mediterranean basin. The assessment of the historical genetic relationships between Spanish sheep breeds using the molecular co-ancestry analysis revealed higher values of *f_ij_*, ranging from 0.237 to 0.297, and highlighted the different ancestral genetic origins of the Iberian sheep [51]. The lower values revealed in our study between the sheep breeds from the two Mediterranean coasts, and reflecting the between-sheep ancestors’ genetica relationships at the moment of their separation, represented a very useful tool that provided reliable evidence on the history of the analyzed Mediterranean sheep groups.

#### 3.2.3. (D_S_) and (δµ)^2^ Genetic Distances and Relative Neighbor-Joining Trees

The pairwise genetic distances, Nei’s standard distance (D_S_) and (δµ)^2^, specifically developed for microsatellites analysis, were presented in Table 5. The performance of the Ds is proven for fine-scale population differentiation analysis, whereas (δµ)^2^ is performed for studying the relatedness among very distinct populations and estimating evolutionary times [68,69]. The highest values of (δµ)^2^ were revealed between APP and DM, and between DM and Venetian breeds, highlighting their very ancient divergence. Meanwhile, the highest Ds distances were reported between the Venetian breeds and the genetically closest group formed by Maghrebian breeds and APP. In fact, the highest (δµ)^2^ distance is displayed between DM and APP (6.609), in contrast to their moderate genetic differentiation recorded in the Ds distance (0.298). The highest Ds distances (0.461 to 0.585) were reported between the Venetian breeds and the APP–Maghrebian breeds group. The distance between APP and the more recent (modern) Maghrebian breeds further decreased (0.213 < Ds < 0.275), reflecting a more recent gene flow practiced between sheep in the close part of the Mediterranean separating the center/South Italy from the nearest Maghrebian countries (Tunisia and Algeria), probably since the Carthaginian and Roman periods.

**Figure 2:**
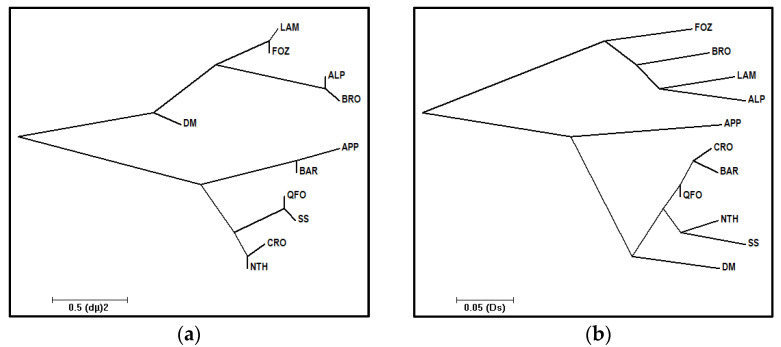
Neighbor-joining trees summarizing phylogeographic relationships between northern and southern Mediterranean sheep based on genetic distances. (**a**) (δµ)^2^ distances; (**b**) Ds distances. Venetian breeds: Alpagota (ALP), Brogna (BRO), Foza (FOZ) and Lamon (LAM); Central Italian breed: Appenninica (APP); Maghrebian breeds: Barbarine (BAR), Western thin-tailed breed (QFO), crossbred BAR × QFO (CRO), D’man (DM), Sicilo–Sarde (SS) and Noire de Thibar (NTH).

The N-J trees were constructed from both genetic distance matrixes (Figure 2a,b). As depicted in both phylogenic trees, the Mediterranean breeds were grouped into two main clusters that did not represent the two proposed groups, the Maghrebian and South European ones. An overlap between these groups was noted in the two trees, while it differed depending on the used distance.

The tree based on (δµ)^2^ demonstrated that the DM breed, of very ancient South Maghrebian sheep, was clustered with the native Venetian breeds, which further argues the ancestral relationship between the North African sheep and the Venetian sheep. The Center Italian APP breed was clustered with the North Maghrebian breeds, suggesting a common ancestral maritime sheep introduction to this Mediterranean corridor and/or a strong gene flow between sheep in this narrow corridor.

In the typology of the Ds’ distances tree (Figure 2b), the DM branch stood out the cluster of the Venetian breeds and linked to the cluster of Maghrebian breeds and APP breeds. The Maghrebian breeds’ group was divided into two further sub-clusters formed by: (1) breeds of northern Maghreb, presenting known and recent genetic relationships reflected by the grouping of BAR, QFO and their crossbred CRO, and the grouping of SS and NTH, both presenting a recent European gene pool introduced at the beginning of the last century and; (2) the South Maghrebian DM breed was isolated in a separate branch, reflecting the genetic drift of this breed argued by the absence of recent genetic exchange with the remaining Maghrebian breeds. The genetic closeness of these geographically closed analyzed Mediterranean breeds can be observed in the study of Ciani et al. [70], where APP and Laticauda, the Italian fat-tailed breed derived from the Maghrebian fat-tailed sheep, clustered together in the same sub-branch. In another study of Italian sheep breeds on a world-wide scale [71], the grouping in the same cluster of the fat-tailed Laticauda with the remaining South-West Italian Bagnolese, the Sicilian and the Sardinian breeds, and in a second cluster, the North Italian breeds, including the Venetian ALP breed, with an intermediate branch position of the center Italian APP, further revealed the genetic closeness of these Mediterranean breeds and their shared genetic background. Furthermore, in a world-wide sheep analysis, Kijas et al. [20] highlighted the genetic closeness of the Mediterranean sheep basing on D_R_ distance, suggesting a major migration route starting from the domestication center (South-West Asia) to the Mediterranean region.

In the case of the Venetian sheep breeds’ phylogeny, the (δµ)^2^ based tree (Figure 2a) demonstrated that the sub-cluster of the Venetian breeds has been split into two subgroups. The first subgroup was formed by LAM and FOZ breeds, highlighting their shared origin. In fact, an ancient contact between LAM and FOZ breed, also named Vicentina, was known during transhumance in the Belluno region (Asiago Plateau in northern Italy) and was considered as the cradle of origin of these breeds [72]. The second subgroup was formed by ALP and BRO breeds. This grouping was not revealed between the same Venetian breeds analyzed by Dalvit et al. [40]. The Ds’ distances clustering revealed that the Venetian group has been separated into four distinct and long sub-branches, which highlighted the recent isolation and discrimination of these breeds after their remote relatedness, as demonstrated in the (δµ)^2^ based tree. These findings put forth fully argued the molecular co-ancestry and gene flow results.

#### 3.2.4. Genetic Structure Analysis

**Figure 3:**
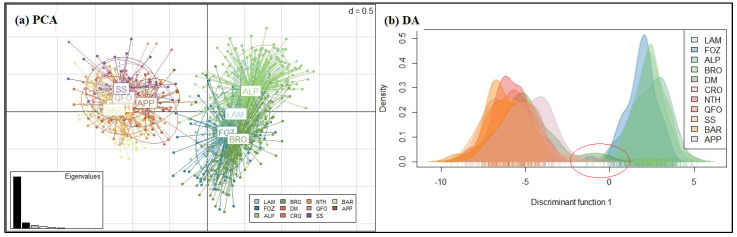
Discriminant analysis of principal components. (**a**) PCA analysis; (**b**) discriminant analysis between the different Mediterranean breeds. Venetian breeds: Alpagota (ALP), Brogna (BRO), Foza (FOZ) and Lamon (LAM); Central Italian breed: Appenninica (APP); Maghrebian breeds: Barbarine (BAR), Western thin-tailed breed (QFO), crossbred BAR × QFO (CRO), D’man (DM), Sicilo–Sarde (SS) and Noire de Thibar (NTH).

**Figure 4:**
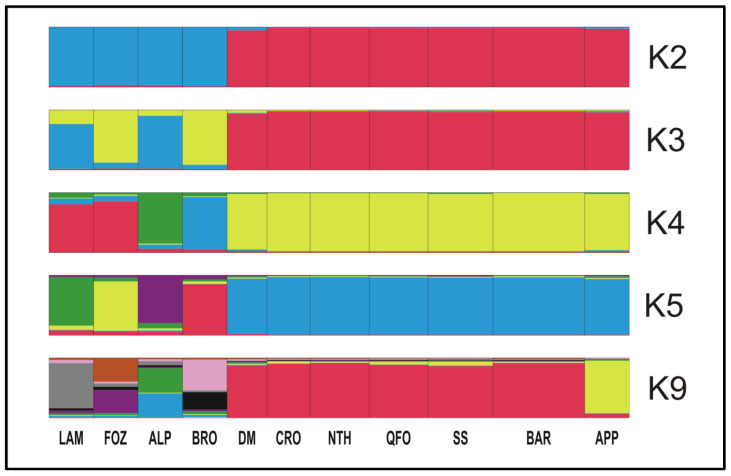
Estimated population structure of the analyzed Mediterranean breeds depicting population membership proportions of each cluster. Venetian breeds: Alpagota (ALP), Brogna (BRO), Foza (FOZ) and Lamon (LAM); Central Italian breed: Appenninica (APP); Maghrebian breeds: Barbarine (BAR), Western thin-tailed breed (QFO), crossbred BAR × QFO (CRO), D’man (DM), Sicilo–Sarde (SS) and Noire de Thibar (NTH).

The phylogenetic results, confirming the usefulness and the performance of the two different scales’ genetic distances (δµ)^2^ and Ds, demonstrating respectively the very ancient and more recent population relationships, are supported by the populations’ structure analysis computed using the discriminant analysis of principal components (Figure 3) and the Bayesian clustering analysis (Figure 4).

Since the discriminant analysis (DA) summarizes between-group genetic differentiation, while minimizing the within-group variation [56,73], we computed a PCA analysis that highlighted the within-group variation [56]. The PCA (Figure 3a) revealed a strong differentiation of the North African–APP sheep group from the Venetian sheep group, demonstrated both a high within-group genetic variation. However, the DA (Figure 3b), which maximizes the separation between groups, clearly revealed the interference, marked by the red ellipse in the *x*-axis l in Figure 3b, between the DM’s density plot (dark green in Figure 3b) and the Venetian breeds plots (the blue and light green plots). In fact, the DA adequately displayed the differentiation of the analyzed Mediterranean sheep, proving the closeness of the center Italian APP sheep and the Maghrebian breeds and the genetic differentiation of the Venetian breeds and their relatedness with the DM south Maghrebian breed.

The Bayesian clustering analysis of the Mediterranean sheep breeds was investigated assuming a number of expected subpopulations (K) varying from 2 to 16 in the whole data. According to the Evanno et al. method [58], it was assumed that K = 4 is the most likely number of ancestral populations. Figure 4 illustrates the clustering at K = 2, 4, 5 and 9; the individual number of Venetian breeds is reduced to 30 per breed to better visualize the whole samples. At K = 2, the Mediterranean breeds were separated into 2 clusters: The Venetian breeds founded the first cluster (blue color), and the Center Italian APP, the SS—resulting from the two Tyrrhenian sheep: Sicilian Comisana and Sardinian Sarda—composed the second cluster (red color) with the native Maghrebian breeds (DM, NTH, QFO, BAR and the crossbred CRO). This result is well supported by the grouping illustrated by the phylogenetic tree based on Ds distances (Figure 2b) and the DAPC analysis (Figure 3a,b). Moreover, the proportion of the ancestral population gene pool of the of Venetian sheep, shared with DM Maghrebian breed (4.8%), which is obviously more important than that shared with the Italian APP breed (2.5%), illustrated the existence of the very ancient relationship between the two ancestors of these two groups, already revealed by the values of the coefficient *f_ij_*, and the phylogenic tree based on (δµ)^2^. This shared gene pool could go back to a common migrant ancestor of the domesticated sheep migrating westward from the center of domestication and/or a later gene flow from the North African Neolithic sheep to the Italian Peninsula. At K = 3, a cluster including the BRO and FOZ breeds was differentiated from the cluster including the LAM and ALP breeds, as revealed in the Ds distances-based tree. At K = 4, the most likely assumed clustering, the Venetian breeds, were split into three groups: ALP and BRO formed their two distinct clusters and LAM and FOZ formed a unique group as revealed by the (δµ)^2^ phylogenetic analysis, highlighting their shared origin during transhumance in the Belluno region [72]. At K = 5, four distinct genetic components corresponding to the actual Venetian breeds are observed with a visible, even low, admixture between them, reflecting their shared remote gene flow before their recent differentiation. With the increase in the number of assumed K, sub-clusters were observed within each Venetian breed, while the Maghrebian breeds and APP maintained the previous clustering as a unique group that was maintained until K = 9, where APP was clearly distinguishable from the Maghrebian breeds.

**Figure 5:**
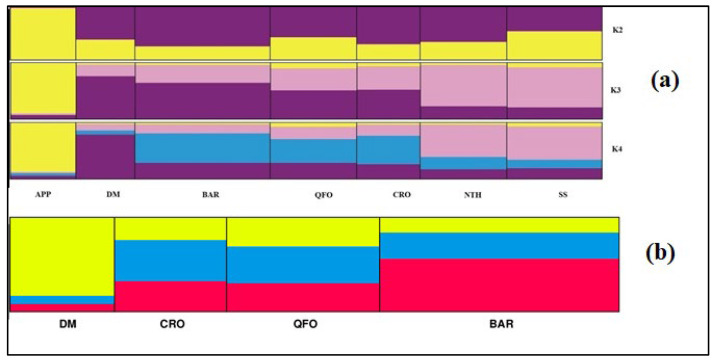
Estimated population structure depicting population membership proportions of each cluster of (**a**) the group of center Italian APP and Maghrebian breeds at K = 2, 3 and 4 (best assumed K); (**b**) Maghrebian native breeds at K = 3 (best assumed K). Central Italian breed: Appenninica (APP); Maghrebian breeds: Barbarine (BAR), Western thin-tailed breed (QFO), crossbred BAR × QFO (CRO), D’man (DM), Sicilo–Sarde (SS) and Noire de Thibar (NTH).

Details of the genetic structure for the group composed by APP and the Maghrebian breeds were computed after removing Venetian breeds as depicted in Figure 5a at K = 2, 3 and 4 (the most probable number of population clusters as revealed by the ΔK method of Evanno et al. [58]). At K = 2, the APP appeared in a distinct cluster with a high membership assignment (92%), while a composite cluster hosted the Maghrebian breeds demonstrating varying memberships of the APP gene pool (64.6% of the SS direct descendants from Comisana and Sarda Italian breeds; 48.5% of the QFO representing the Algerian Ouled Djellal breed; 39.8% of NTH crossbred of Ouled Djellal and French Merino d’Arle; 27.3% of the fat-tailed BAR breed and 23.5% of the Moroccan DM breed. This considerable presence of the North Mediterranean APP gene pool in the South Mediterranean Maghrebian breeds, especially in the Moroccan DM breed, which is the most geographically distant from Italy, goes back to the shared genetic background of the first Neolithic westward migrant sheep from the Fertile Crescent. The percentage of this common part in the remaining Maghrebian breeds would be proportional to the subsequent exchanges of genes between the two northern and southern coasts of the Mediterranean. In fact, two westward sheep ancestors would have reached the Mediterranean basin: the yellow color (major gene pool of APP and SS) represents the first ancestor introduced by sea to the northern and southern Mediterranean shores and the purple color represents the second ancestor that arrived in the Maghreb mainly by land via Egypt and Libya. At K = 3 (Figure 5a), the admixture origin of the Maghrebian breeds is clearly defined as a miscegenation between two ancestral populations (probably one coming by sea and the other one by land), while the APP was assigned to one ancestral cluster maintained in the increased assumed K. The most likely number of ancestral populations (K = 4, Figure 5a) highlighted the phylogeographic patterns of the analyzed Mediterranean breeds. In fact, apart from the ancestral population of the APP (yellow color), three ancestral populations appeared in the miscegenated genome of the Maghrebian breeds. In chronological order, the purple color would be the first ancestral sheep that populated North Africa at the Neolithic; the blue color, present with the highest proportion in the BAR fat-tailed breed, should represent the second ancestral population that had invaded North Africa and the Maghreb during a second wave of migration from the domestication center and that would be a fat-tailed sheep; and finally, the pink color present with a high proportion in SS and NTH genome would represent the south European gene pool, since the respectively Italian and French gene pools’ introgression in SS and NTH were known. To better argue our hypotheses, a deeper structural analysis was carried out with only the native Maghrebian breeds: South Maghrebian DM, Algerian QFO/Ouled Djellal and Tunisian BAR. Figure 5b depicted the result at the optimum number of ancestral clusters, with K = 3 inferred from ΔK method [58]. The miscegenation of the three ancestral populations with different proportions contributing to these native North African breeds’ genetic heritage was clearly demonstrated. The first ancestral population (yellow color in Figure 5b) specific of the DM breed should match the first sheep ancestor migrating to North Africa and the Maghreb. The high conservation level of this ancestral gene pool in the DM genome can be explained by the geographical isolation of this breed in the southern oasis of Maghreb (in the northern Sahara board); thus, a genetic drift as a founder effect of this sheep ancestor would have occurred. The second ancestral population (red color in Figure 5b), prevailing in the fat-tailed BAR genome structure, should represent the fat-tailed sheep migrant from the domestication center, reaching the western Mediterranean basin, especially the North African coasts and probably with the Phoenician migration. The third ancestral population (blue color in Figure 5b) represents the South European gene pool introgression, which occurred more recently after the establishment and the miscegenation of the two previous ancestors. This tripolar genetic structure of the Maghrebian sheep can also be revealed in the microsatellites’ analysis of the Tunisian [24], Algerian [25,27] and Moroccan [30] sheep breeds and in the mitogenome analysis of Algerian [34] and Moroccan [32] breeds.

#### 3.2.5. Divergence Time

In order to support the proposed chronological order of the ancestral sheep migrations to the occidental Mediterranean basin, highlighted by the genetic distances and the structural analysis, we calculated the divergence times between the analyzed breeds (Table 6) from E(δµ)^2^ = 2 βτ; where β: mutation rate and τ: time of generation [53,68]. The absolute sheep mutation rate for dinucleotide microsatellites was 1.3 ± 0.5 × 10^−4^ mutations per gamete per locus, as estimated by Crawford and Cuthbertson [74]. However, these authors have interpreted this mutation rate as underestimated, because mutations were not detected and may have been considered as existing alleles. Furthermore, this study mentioned that more polymorphic markers have a higher mutation rate than the less polymorphic ones. Recent studies demonstrated that microsatellite mutation rates are correlated with alleles’ lengths and suggested an even higher rate of about 10^−3^ per meiosis for mammalians [75,76]. Elfawal et al. [77], studying the phylogeny of Egyptian sheep breeds, have used a mutation rate of 1.2 × 10^−3^. This value was defined in Iranian sheep by Esmaeilkhanian and Banabazi [78], as the mutation rate of the locus Oar FCB 304 belonged to the panel of our study. As all the 17 microsatellite markers used in this study were highly polymorphic and had high allele lengths (Table 2), we used the mutation rate of 1.2 × 10^−3^ to estimate the divergence time of the analyzed breeds. An average generation interval of 3.5 year/generation between European [79] and Tunisian [80] sheep was used to calculate the absolute divergence dates of the analyzed breeds in a number of years. The highest value of the estimated divergence time was approximately 11,000 years ago, calculated between the Moroccan DM and the APP center Italian breed. The divergence period between the DM and the Venetian breeds was estimated to have occurred in the next centuries later on (from 10,736 to 9392 years ago). These estimated dates indicated that these divergences would have occurred in the domestication center between the ancestors of these breeds just before their westward migrations. The ancestor of APP, diverged 11,000 years ago, would been the first immigrant reaching the western Mediterranean basin, certainly by sea, followed by the ancestors of the Venetian breeds and the South Maghrebian DM breed. These estimated dates, coinciding with the earliest phase of the Neolithization process, could be concurrent to the first westward migration waves of the domesticated sheep from the Fertile Crescent. In fact, the earliest sheep westward migration wave, 11,000 years ago, was mentioned by Chessa et al. [81] and Barbato et al. [82]; the starting of the migration by sea of domesticated sheep (*O. a.*) to Cyprus has been proven since the 11th millennium [83].

Since the divergence time between DM and the remaining Maghrebian breeds is the oldest within the group separation (approximately 5000 years ago), the DM should have an ancestral relationship with them, as revealed by the structure analysis (Figure 5a,b). This divergence could be caused by a past event that happened 5000 years ago, which should be considered the mid Holocene desertification shift event of North Africa. Archeological, historical, climatical and morphological arguments supporting this hypothesis will be presented in the following section. The divergence time between the unique and the most native fat-tailed sheep of the Maghreb (BAR) and the remaining native thin-tailed groups (APP, Venetian breeds and DM) ranged from approximately 6900 to 5000 years ago. This period is contemporary to the divergence time between the thin- and fat-tailed sheep class developed in the Fertile Crescent (5500 years B.P.), estimated by Moradi et al. [84]. The most recent divergence time (about 3500) between both Mediterranean coasts’ sheep was noted between the Italian originated breed, the SS (or its ancestors Comisana and Sarda) and the BAR. This divergence may coincide with the Phoenician period, probably considered as the introduction of the major wave of fat-tailed sheep to North Africa, especially to Carthage (actual Tunisia). Moreover, the unexpected recent divergence time (about 1400 years ago) between the fat-tailed BAR and the thin-tailed QFO, originating from the Algerian Ouled Djellal breed, may be explained by an unique shared genetic background represented by an ancient miscegenation of these two fat- and thin-tailed sheep classes; however, later on, the crossing with the thin-tailed sheep (from Europe or from the Sahara), allowed the Ouled Djellal breed to recover its thin tail phenotype, which was considered as a dominant character [84]. It is worth mentioning that this divergence time (about 1400 years ago) between the Tunisian specific fat-tailed sheep and the Algerian specific thin-tailed sheep is contemporary to a second wave of fat-tailed sheep reintroduced from the Middle East. This recent gene flow would have contributed to diluting the preexisting genetic structure in BAR and the dominance in its genome of the fat-tailed ancestral gene pool (red color in Figure 5b). The divergence time between the Venetian breeds, ranging from 2900 to 1500 years ago, would have started in the Roman times and continued at the medieval period. The within-Venetian breeds’ differentiation could coincide with the beginning of fine wool sheep selection in the occidental part of the Mediterranean, having started between the Roman era and medieval period [70].

### 3.3. Phylogeographic Patterns of the Sheep Migration Waves’ Westward Domestication Center Inferred from Genetic Data and Supported by Archeological, Historical and Systematic Data

The present genetic study constitutes an inference on the evolutionary history of sheep from both the Northern and Southern shores of the occidental Mediterranean basin, considered as the main and the earliest migration road from the domestication center [83]. The migration and past gene flow had a key role in shaping the genetic structure observed in the modern-day breeds [20]. Analyzing primitive and native sheep breeds, which have not been under powerful human-driven selection that can overshadow the phylogeographical signature, allows one to faithfully reflect the geographical origin, the past gene flow and admixture pattern between populations [71]. The congruence of the results between the adopted approaches (gene flow, molecular co-ancestry, remote and recent genetic distances, phylogenetic trees, DAPC analysis and Bayesian clustering) on one hand, and their complementarity with archeological, systematic, climatic, historic data and between breeds of phenotypic similarity—that we required as a result of the prompting made by our genetic results—on the other hand, leads us to propose a faithful scenario reconstructing the phylogeography of sheep in the occidental Mediterranean basin since the early Neolithic. In addition, genetic data of the westward human lineages’ migration from the Fertile Crescent to the occidental Mediterranean Basin, since the early post glacial waves until the modern times [85,86], will be presented to further support the present phylogeographic findings of the sheep, since these human lineages should introduced with the sheep from the earliest domesticated to the most evolved forms. These multidisciplinary data should further support the following proposed scenario that would perfectly reflect the westward sheep migration waves from the Fertile Crescent, respecting a chronological order presented as successive migration phases. The different performed approaches revealed an ancestral relationship between the sheep of both the Mediterranean coasts. However, this ancestral relatedness is scheduled to different anterior periods, which we will describe as phases divided into more than one wave.

#### 3.3.1. First Migration Phase

During the first migration phase, three of the first sheep migration waves from the Fertile Crescent that reached the studied closest part of the occidental Mediterranean basin, following both maritime and land routes, could be concluded:

1. The first wave of this earliest migration phase was not highlighted by the present study. It would have occurred in the precedent Millennia, since the earliest divergence time was revealed in the present study between the analyzed Mediterranean breeds remote to 11,000 years ago. This first wave could correspond to the earliest form of the first domesticated sheep in the Fertile Crescent in the early Holocene before 12,000 years ago, which was very close to the wild ancestor (*O. g.*). This earliest domesticated form migrated via the Mediterranean Sea and occupied Cyprus since 12,000 [87], and later on, the Tyrrhenian area (Corsica and Sardinia), where a return to the feral form occurred, leading to, respectively, the Cyprus mouflon (*O. g. ophion*) and the European mouflon (*O. g. musimon*) of Corsica and Sardinia [87,88]. The presence of a second post glacial human occupation was highlighted in the Tyrrhenian islands and in the south-western Europe in the early 12th millennium B.P., described as the Holocene human re-expansion [86,89] induced by climate warming, which favored the early Holocene seasonality just preceding the Younger Dryas and the high fire frequency events [90]. Recent studies demonstrated that sheep domestication was pushed back to the late Epipaleolithic (13th–12th millennium B.P.), allowing for the first domesticated sheep with wild morphology [2,91]. This sheep was a hairy and short-tailed sheep, such as the wild ancestor and the remnant mouflon. The findings of Vigne et al. [83], highlighting the early Mediterranean transportation of mammals by boats starting in the 13th millennium and later specialized by human voyagers, supported this earliest migration wave of domesticated sheep with wild morphology, which led to the establishment of the Mediterranean mouflon. This first wave seems to concern only the northern Mediterranean rim, since:

- An early post last glacial occupation of southern Europe and especially Tyrrhenian island, by Mesolithic migrant human populations, which moved to the continent from the Near East, was evidenced at around the 12–11th millennium B.P. [92,93,94,95];
- No evidence of mouflon and short-tailed sheep was noted in North Africa;
- Waves of human population from the Middle East to North Africa occurred 15,000 and 9000 years ago [85], before the initiation of sheep domestication and after the first appearance of the morphologically domestic sheep with a long tail, out of the domestication center in the Mediterranean Cyprus, around 10,500 years ago [2,83,95];
- The climate conditions in the 12th millennium’s North African Paleolithic noted a relatively dry episode [96,97,98] that could have prevented the introduction of this westward Middle Eastern Human wave, with their first domesticated wild sheep (mouflon), where the Northern Mediterranean basin conditions were more favorable.

Another explanation of the absence of mouflon from the North African region, in addition to its extinction from the majority of the southwest Europe regions except the Tyrrhenian Islands, would be the fire activities’ effect recorded in both Mediterranean coasts, preceding from the drier North Mediterranean side that was favorable to a high fire regime in the 8th millennium [97,98]. A further analysis including the Mediterranean mouflon might be useful for a more complete overview of the phylogeographic pattern of sheep since the first domestication phase.

A land migration of this first sheep ancestor, via the Danube reaching central and northern Europe, led to establishment of the primitive North European short-tailed sheep group, from which survived the most primitive Soay sheep and has been extinct the Centre European Turbary sheep (*O. a. palustris* or *O. a. studeri*) discovered in the Swiss Neolithic Lake Dwellings [99]. This short-tailed primitive sheep returned to a feral form [100] and is considered with the Mediterranean mouflons as the remainder of this first immigrant wave reaching Europe [81,101];

2. The second migration wave, perfectly highlighted by the present sheep genetic analysis, is considered as the first Neolithic westward introduction of phenotypically domesticated sheep. This migration wave could have been represented by the three contemporaneous migrant sheep that are the ancestors of the center Italian APP sheep, the Venetian sheep and the DM sheep of the Maghreb. The very ancient relatedness between these sheep was prove by the (*f_ij_*), and the (δµ)^2^ distances and the shared ancestral gene pool, especially between the DM and Venetian breeds, depicted by the structure analysis, and mainly by their divergence, approximately between 11,000 to 9000 years ago (Table 6). These three ancestors, as depicted by the phylogenetic trees (Figure 2), diverged from a common ancestor (first node), which would be the earliest domestic-like sheep and the descendant of the morphologically wild sheep domesticated in the Fertile Crescent and described above in the first wave. Our revealed genetic divergence faithfully coincided with the management step of the morphologically domesticated sheep, which replaced the first domesticated sheep with a wild morphological form in the domestication center mentioned by Zeder [2,95] and Vigne et al. [83]. In fact, these scholars and Zeder [91] mentioned that the most important phenotypic changes—mostly in horn and body sizes and in shape, such as changes in rachis morphology influencing the tail length—had been noted over a 1000 years ago, following the 12th millennium, which reflected a change in the management practices of animals. It is worth mentioning that actual Mediterranean sheep breeds belong to the long-tailed group and migrated westward from the Fertile Crescent during this second wave of the first migration phase after these mentioned changes, especially the rachis morphology change. In fact, tail length, which is a strongly inherited trait and academically interesting feature in sheep classifications [102], is based on the number of coccygeal vertebrae. The mouflon presented a short-tailed vertebrae (no more than 11); the North European short-tailed primitive sheep group, such as the Soay breed, had 8 to 10 vertebrae; and the long tailed sheep group had 16 to 18, reaching to 24 vertebrae in the tail [101,102]. This progression in the tail length reflects the sheep evolution morphology after the domestication effect. Otherwise, recent structure analysis, including mouflon, primitive breeds and modern Mediterranean breeds, demonstrated a clear distinction (at K = 2) between the two ancestral populations of mouflon and domesticated sheep [21,70,82]. Barbato et al. [82] and Ciani et al. [70] highlighted the first differentiation of the primitive short-tailed Soay sheep from the morphologically long-tailed domestic sheep, which faithfully argues our hypothesis of two distinct waves in the first phase of the westward migration of sheep, in where the short-tailed Soay breed would occupied an intermediate position between these two waves. Consequently, the three contemporaneous ancestors of this second wave should have been derived from a common long-tailed domesticated sheep ancestor, which was already evolved in the domestication center and then migrated westward, leading to its recent descendants: The Venetian breeds, the central Italian APP and the Maghrebian DM breed.

**Figure 6:**
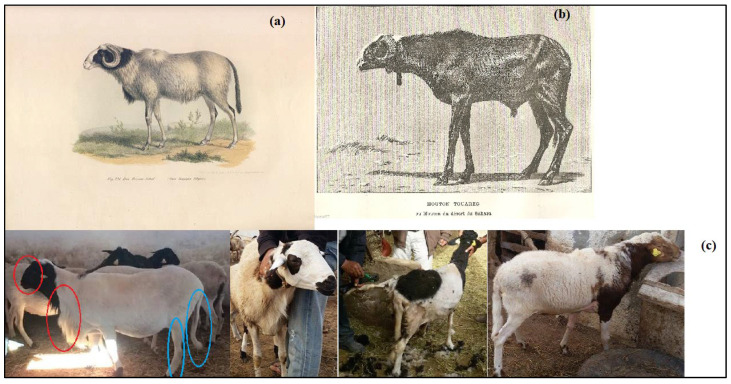
Morphological data providing the characteristic of the *O.* *a. longipes* subspecies. (**a**) Fitzinger’s lithography of the Neolithic Maghrebian sheep ancestor; (**b**) Tuarg/Touarg sheep of Northern Sahara; (**c**) actual Maghrebian D’man (DM) breed; ellipse highlighting the Roman nose, long thin tail and leg, hairy man and mixed hairy and woolly coat.

The archeological and anthropological analysis highlighted the earliest westward Neolithic human migration that we detailed in the following and faithfully coincided with the proposed scenario for the second wave migration:

- The first ancestor, from which derived the central Italian APP sheep diverged from the Maghrebian DM breed since 11,000 years ago, would be the first Neolithic sheep migrated by maritime road from the domestication center, reaching South Europe, going through Cyprus, and then the Greece islands to reach the Apennine peninsula—the cradle of the APP breed—where the spread of the earliest Impressa Neolithic group characterized by the sheep breeding dominance at the 9th millennium was revealed [16,86,95]. This early Neolithic sheep presence in the Italian peninsula was achieved or reduced probably due to the 8.2 cal ka climatic cooling event and the maximum fire activity noted in this region [86,95,97]. The APP’s ancestor would have migrated, after these unfavorable conditions, to the southern Mediterranean coasts, where the humid climate of the green North African begins [97], as evidenced by the high molecular co-ancestry and gene flow values between APP and the Maghrebian breeds—greater than the values between APP and the Venetian breeds—and their shared common ancestral populations of up to nine assumed ancestral populations (K = 9). This was depicted by the structure analysis and the phylogenetic trees (Figure 2a,b) clustering together the APP and Maghrebian breeds. The genetic relatedness between the Maghrebian and the south-western European breeds was highlighted by Kandoussi et al. [32], who revealed the closeness of the Moroccan and the Italian sheep groups and suggested a south Italian–Tunisian introduction route of Neolithic sheep from the Middle East domestication center. The hypothesis that the APP’s ancestor has contemporarily directly reached the southern Mediterranean rim might not have to be excluded, since a strong molecular co-ancestry relatedness had been revealed between the APP and Maghrebian breeds, in addition to the next gene flow reported between them. However, the absence of a sheep trace in North Africa in the 9th millennium can be explained by the geoarchaeological data revealing a dry North African climate in this period [97] and, thus, avoiding the early Neolithic expansion in the southern Mediterranean area, and/or probably due to the absence or the scarcity of zooarcheological research highlighting this presence in the southern rim comparatively to those of the northern Mediterranean, as proposed by Zeder [95], explaining the lack of evidence of the earliest Neolithic trace in North Africa before the 8th millennium B.P. The two remaining ancestors, from which the Venetian breeds and Maghrebian DM breed were derived, diverged between the 11th and the 10th millennium (Table 6) from a common ancestor, as revealed by the molecular co-ancestry (*f_ij_*) relatedness and the (δµ)^2^ distances-based tree, and was migrated by two different land roads;
- The second ancestor is the Venetian breeds’ ancestor. It was diverged from the Fertile Crescent ancestor since the 11th millennium (10,736 years ago, Table 6), and went to South Europe by the northwestern overland road known as the Danubian route, considered as a gateway of the Europe Neolithization [103]. The very ancient remote relatedness between the Venetian breeds before their recent differentiation revealed by the (*f_ij_*) parameter strongly supported this idea and was well proven by the two phylogenetic trees (Figure 2a,b), as well as the PCA, DAPC and Bayesian structure analysis. This ancient genetic relatedness was supported by the historic data revealing that by the Danubian, immigrant farmers—Danilo groups from Balkans—introduced domestic animals into Istria and northeastern Italy. This northern land introduction to the actual Venetian region was mentioned in addition to the earliest and speeder migration by the Impressa group, which introduced agriculture to the southern and center-west of Italy, probably introducing the ancestor of APP. These two different introduction roads of agriculture into Italy explained the genetic differentiation of the two ancestral populations of Venetian breeds, and APP was described as the first and the second ancestors of the westward second sheep wave. The two different sheep introductions to the Italian Peninsula, the Mediterranean maritime route and the land route via the Balkans were highlighted by Ciani et al. [21].
- Exploring the systematic data of the Venetian sheep, we discovered, unexpectedly, the systematic classification “*O. a. sudanica* Sanson” of the Alpine sheep group, from which the Venetian and Bergamesca breeds were derived [104,105]. This classification is relative to an ancient supposed Sudanese origin of the Alpine sheep based on the phenotypic similarity between old Alpine sheep and Sudanese native sheep [105,106,107]. This classification would explain the noticeable level of the molecular ancestry identity between the Venetian sheep and the North African DM sheep or its ancestor, described as the third ancestor of this westward second sheep wave;
- The third ancestor is the direct ancestor of the DM breed due to the high conservation level of its non-miscegenated ancestral gene pool. This ancestor would have diverged in the Fertile Crescent since the 11th millennium. It would have taken the southwestern overland road, via the Suez Ishim, Egypt and Libya, in order to reach Tunisia, Algeria and Morocco. The DM seems to be the legacy of this ancestor, and is perfectly conserved after its genetic drift and isolation in the northern Sahara oasis. Our hypothesis based on phylogenetic inference is strongly supported by historical and archeological data. In fact, the first ancestral sheep reaching North Africa was depicted in an old Egyptian monument and in the Maghrebian Cavern, most often apprehended through engraved or painted representations as a hairy sheep with long tail and legs [108,109]. Basing on sheep representations found in Egyptian, Libyan and Atlas monuments (Saharan Algeria), this ancestry was respectively identified as *Ovis longipes aegyptiaca* for Egyptian ancestry sheep and, for Maghrebian ancestry sheep, as *Ovis longipes libyca* Fitzinger (1860) or “Fezzan sheep“ defined by Fitzinger [110] using the first representation of this ancestral sheep found in Fezzan in Libya (Figure 6a). As mentioned by Joleaud [111] and Camps [108,109], these subspecies were regrouped by Fitzinger (1860) under a unique systematic classification as *O. a. longipes* Fitzinger (1860), in relation to the very long legs and tail of this group of the first ancestors of hairy domestic sheep. The gap of the archeological evidence of this ancestral sheep in Tunisia was filled after the recent findings of Ben Naser [112,113] discovering the sheep (*O. a. longipes* Fitzinger) representations, in the Central Tunisian caves of Jbel Ouesslat, considered as the northernmost currently known engravings on the rocks of the Maghreb, dating from the early to middle Holocene and illustrating both Capsian and Neolithic periods (8200 to 6000 years ago). Until our findings, it was assumed that this ancestry was extremely extinct from North Africa (Egypt, Libya, Algeria and Morocco) and that its descendants still existed in the Sahel and Sub Saharan region from Mauritania to Chad, as the Tuareg sheep of the Saharan Algeria (Figure 6b) and West African sheep as the Dwarf of Nigeria and Cameroon [108,109,111]. The phylogenetic data from our genetic analysis led us to investigate the archeological, historical and morphological data to confirm our hypothesis, considering the actual native DM breed of the Maghreb as the last representative of the first ancestral sheep reaching North Africa in the first Neolithic immigration wave. As mentioned by Tourte [114], in 1068, the Arab historian El Bekri described the sheep of the western Sahara as a hairy sheep with long legs and tail named “Dammanian sheep”; this nomenclature is considered as the Arabic name of the subspecies *O.* *a. longipes* Fitzinger. In fact, the actual name “D’man” or “Damman” (DM) of the Maghrebian breed was derived from this Arabic nomenclature of the Sub Saharan hairy sheep. Consequently, the DM is the last representative of this subspecies that is supposed to be extinct from the Maghreb. At a morphological level, we discovered a perfect congruence between the current DM breed and the first ancestor of the North African sheep, faithfully reproduced on the engraved or painted representations in Maghrebian caves of the Saharan Atlas dated from the early Neolithic. In 1860, Fitzinger [110] finely reproduced the features of this ancestral sheep of North Africa dated from the earlier Neolithic period in a figure entitled “Das Fezzan-Schaf. *O. a. longipes libyca*” (Figure 6a). We illustrated the fine compatibility—big size, very long legs and tail, presence of man, roman nose (a slightly domed chamfer) and mixed hairy and woolly coat between Fitzinger’s lithography of “Fezzan sheep” (Figure 6a) and the current DM sheep of the Maghreb (Figure 6c).

The morphological character of the Fezzan sheep seems to be perfectly inherited by the DM sheep and by other actual western African sheep (Tuareg and Dwarf), as far as current East African thin-tailed and mixed coat hairy/woolly sheep present in Sudan Desert (Watish, Shugor and the Dubasi) that actually extend eastwards into Eritrea and westwards into Chad, which aresupposed to be originated from the very old and extinct Egyptian thin-tailed ancestry sheep [115]. Some of these characteristics, especially the relative big size, the roman nose and the colored hairy areas, are still observed in some Italian Alpine breeds [106]. In addition to the ancient genetic relatedness that we have revealed between the Venetian breeds and the DM, the systematic data classified the Venetian breeds under *O. a. sudanica*, belonging to the subspecies *O.* *a. longipes* Fitzinger [104,105,106,107]. The question is, why the legacy of the first immigrant thin-tailed sheep reaching Africa has completely disappeared from North Africa and has survived in the Sahel and sub-Saharan region from Eritrea to Mauritania. The paleoclimatic data associates the later observation to the transition of Northern Africa, from the “green Sahara” during the early Holocene African “Humid Period”, to the largest warm desert today [96,116]. The present Sahara Desert was, in the early Holocene (9000–6000 years B.P.), a savannah ecosystem with a favorable climate supporting pastoral activities and nomadism. In the middle Holocene circa 5000 years B.P., a dramatic climatic shift triggered an abrupt process of desertification of North Africa, which was completed within a few hundred years [116]. The desiccation of the Sahara has forced a southward movement of North African herdsmen together with their livestock (goat and sheep) to reach the Sahel [117,118]. During this migration event, the North African thin-tailed ancestral sheep *O. a. longipes* Fitz would have left North Africa and would have occupied the Sahel and Sub Saharan regions. Until the present genetic investigation, it was thought that this desertification eradicated the majority of natural archives [116] and that this ancestral sheep had completely disappeared from North Africa (Egypt, Libya, Tunisia, Algeria and Morocco). However, the presence of the ancestral gene pool of the *O. a. longipes* in the actual North African sheep breeds, illustrated in Figure 5a,b, highlighted the miscegenation of the remaining population of this ancestor with two other ancestral sheep that reached later the Occidental Mediterranean basin, as we detailed in the second migration phase scenario. The presence of the ancestral DM’s gene pool in the genome of actual Tunisian, Algerian and Moroccan native sheep breeds is clearly visualized in the structure plots of recent genetic investigations [25,27,30,119], though it was not interpreted as an ancestral breed. Kandoussi et al. [32], using a maternal heritage marker, revealed that the DM breed presented a purely Moroccan genome with the ancient expansion time (from 8600 years ago) to the Maghreb region, strongly corroborating our findings. Moreover, the Sahara desert, which transformed in a veritable geographic barrier to human and livestock movements between the North Africa and Sub-Saharan region, would have prevented the return of *O. a. longipes* northward to the actual Maghreb and Egypt. Thus, this first ancestral sheep, shipped southward of the Sahara, should have been confined and isolated from more recent Maghrebian breeds, except the DM breed and other north Saharan breeds not included in this study, known as the Algerian Sidaoun and Hamra [25]. These breeds seem to have been surviving and drifting in an isolated oasis area in the northern border of Sahara, or came back later with more recent migrations of the nomadic Pastoralist. The actual “Tuareg” or “Touareg” human community, maintaining pastoral and nomadic lifestyle between the Sahel and Central Sahara region, since the earliest Neolithic time and, raising a very old sheep breed known also as the Touareg sheep (Figure 6b), should be the current representative population of the human Maghrebian ancestor, having attended the first introduction wave of sheep in North Africa, and the later migration southward imposed by the desertification of the middle Holocene [7,120]. This last study, based on neutral, maternal and paternal human DNA analysis, demonstrated that the Tuareg human community, descending from the Libyan human ancestral community of Fezzan, which attended the first Neolithic wave from the Fertile Crescent (9000 years ago), was entrapped by the desertification into the Sahel belt after some Millennium (5000 to 3000 years ago). The common origin between the Tuareg human population and the eastern Sudanese Beja population was also mentioned by this study [120]. Contemporaneous Neolithic human migration from the Middle East to Tunisia, 9000 years ago, was recently revealed by Elkamel et al. [85]. The high congruence between both the human and sheep ancestors’ history further supports our proposed scenario based on neutral microsatellite markers. In fact, Fazzen sheep (*O. a. longipes libyca* Fitzinger), called “Damman” in Arabic, and having a common origin as *O. a. sudanica*, the ancestor of Venetian breeds, would be the first ancestral sheep to reach North Africa reared by the Berber human population (ancestor of Tuareg) living in the first Neolithic diffusion with pastoralist lifestyles. These ancestral sheep, with their herdsman, were forced to migrate southward after the climate shift causing the North African desertification, as a result leading to both the isolated and drifted human and sheep populations, respectively, known as the current human Tuareg and the actual Tuareg sheep, and/or the DM sheep having the same morphological traits (Figure 6a–c). These two populations, human and sheep, have survived until now in the Sahara without much changes in their lifestyle, as well as in their genetic background, since the earliest Neolithic period.

The morphological features of this first immigration sheep phase can be resumed in the gig size, the presence of darker or colored skin, coarser hair instead of a woolly fleece or a mixed hairy and woolly coat, a shedding/molting ability of the winter coat during the spring and summer months [81], the presence of a long man by mature rams and a short one by some ewes and the presence of roman nose [106]. These primitive characters were present in the extinct sheep *O. a. aegyptica*, *O. a. libyca* and *O. a. sudanica*, and still perfectly observed in the actual DM breed. Furthermore, these primitive characters are present in Tuareg sheep, West African sheep (Dwarf, Djallonke), Indian primitive hairy sheep (Mecheri, Kilakarsal) and Caribbean hair sheep resulting from West African introgression into previously introduced Iberian wool sheep [121], as well as in the American hairy sheep group resulting from the actual crossing of European mouflon with modern wool sheep breeds. In addition, it was proven that the archaeological European Copper Age sheep “Otzi’s sheep” dating to 5300 years ago and discovered near the Austro–Italian border, presented hairy fibers in its coat [122]; thus, it would be a hairy or mixed hairy/woolly sheep. This discovery further justifies the old belonging of Venetian breeds to the *O. a. sudanica* class with a mixed hairy/woolly coat, before its later miscegenation with woolly sheep, which next reached the region [123].

A highly probable miscegenation would have happened between the first ancestor reportedly following the seaward route to the northern rim of the Mediterranean, from which derived the central Italian APP, and the first North African sheep ancestor reaching the Maghreb by land via Egypt and Libya, from which survived the primitive DM breed. The second migration wave of the first phase of the sheep migration in our proposed scenario would have been achieved before the complete evolution of the Neolithic sheep, identified as the long and thin-tailed sheep with a hairy or mixed hairy-woolly coat, to the more evolved woolly and then fat-tailed sheep appearing in the domestication center. This evolved sheep constituted the second phase of the westward sheep migration, which happened starting from the late Copper Age and the early Bronze Age and was continued in historic times.

#### 3.3.2. Second Migration Phase

The second migration phase is represented, in the present study, by the divergence and then the westward migration of the woolly and fat-tailed sheep, the BAR, from the domestication center during the 6th millennium. This divergence was started in the 7th millennium, which revealed the divergence time between the BAR and APP (6943 years ago, Table 6) and concerned the remaining Mediterranean woolly and thin-tailed breeds, QFO, NTH and SS, which diverged from *O. a. longipes libyca* Fitzinger DM’s ancestor between 6759 and 5847 years ago and from the Venetian breeds’ ancestor between 6334 and 3530 years ago, except the more ancient divergence between NTH and BRO, which was 7452 years ago. Overall, our findings revealed that the divergence of the woolly sheep occurred in prehistoric times, beginning in the early Copper Age or Chalcolithic (from the late 7th millennium to the late 5th/early 4th millennium), passing through the Bronze Age (5300 to 3200 years B.P.), ending in the Iron Age (between the early 4th millennium (3200 years B.P.) to the middle 3rd millennium—[2500 years B.P.]). The relative dating of the analyzed breeds’ divergence, forming the second westward sheep migration phase, is very informative because these dates should roughly correspond to the following zooarcheological, ancient climatic change and textile evolution dates. In fact, on the basis of archeological and written prehistoric sources [124,125], the evolution of the first domesticated hairy sheep to the woolly sheep also occurred in the domestication center as late as the late 8th to early 7th millennium, and then migrated westward to the occidental Mediterranean basin during the late Copper Age, and then the Bronze Age to the Iron Age. Driven by the climatic change from the wetter early Holocene to cooler and drier conditions beginning in the 7th millennium [123,126,127], the evolution from the hairy to the veritable woolly sheep would have taken many millennia, after a mutation process due to a retro-gene insertion resulted in graduate coat qualities from the hairy wild ancestor to woolly sheep [128]. This process, thought to have occurred in Mesopotamia during the later Copper Age [123,129], is presumed as the reduction of the hair and Kemp fibers, as the development of the fine under-wool of the winter undercoat did not change seasonally any more, but grew continuously, as well as, finally, the loss of the hair pigmentation [129]. Vila and Helmer [124] emphasized that sheep were kept for their fleece in the Fertile Crescent around the 8th millennium B.P. Later on, the keeping of woolly sheep was systematically practiced on a larger scale and occurred in a fairly narrow time span in the 6th millennium [129]. These later studies [123,129] mentioned that the introduction and spread of woolly fleece-bearing sheep husbandry to prehistoric Europe occurred at the Early Bronze Age (c.a. the 4th millennium), where wool production was established. Sabatini et al. [130] mentioned that the wool production in the Bronze Age in prehistoric Europe could have happened through the introduction of new foreign woolier animals that might be mixed in with the local sheep. The introduction of new woolly sheep breeds from the near East to the Italian peninsula was evidenced to since the Copper–Bronze Age transition by the 6th–5th millennia B.P. [11,86,125]. Two wool sheep waves occurred in this second migration phase. The first wave introduced the first woolly sheep with short and coarser fleece in the late Copper Age, and the second wave happened later, in the late Bronze Age and Iron Age, and imported a finer and longer woolly sheep fleece after the involvement of the wool breeding and fiber selection in the domestication center [124,129,131]. In fact, the divergence time between the analyzed Venetian breeds dating from 2928 to 2753 years ago, except the divergence of the FOZ breed that occurred later in the Middle Ages, was related to the last migration wave of finer wool sheep with small-sized animals arrived to the Alpine region. These new sheep populations coexisted together until the Iron Age, with the large-sized animals coming during the Bronze Age wave, still presenting a natural molt in spring [123]. The miscegenation of these successive woolly sheep populations absorbed and diluted the first phase of the sheep gene pool, since no isolation of this first phase ancestor had occurred as was isolated and drifted *O. a. longipes* in the southern Mediterranean rim. In fact, Grömer and Saliari [123] mentioned that the art of breeding sheep with fine and pure white wool in the Northern Italy Veneto region was practiced during the Roman Period. Gleba [125] revealed in Roman written sources that a good fine wool quality of the extinct Altino Venetian sheep was described as superior to the southern Italy Tarentum ancestor of the Merino sheep.

During the same period and in parallel to the evolution from the hairy to woolly sheep, and induced as well by the drying climate change, an evolution from thin- to fat-tailed occurred in the Fertile Crescent [124]. Archeological evidence for the fat-tailed sheep was presented for the first time towards the end of the 6th millennium, and a preference for smaller sheep varieties with fat tail and woolly fleeces was emphasized in Mesopotamia after the increase in urbanization, generating the necessity of large scale production [124]. Written sources evidenced the presence of fat-tailed sheep with the woolly variety between 5200 to 4000 B.P. [129]. These zooarcheological data corroborate with the divergence time of the fat-tailed BAR sheep estimated from our genetic data. The fat-tailed sheep presence in ancient Egypt, classified as *Ovis platyra aegyptiaca,* was largely depicted between 3002 and 2644 B.P. in Egyptian monuments [132]. This fat-tailed sheep would reached Libya 3000 years B.P. [133]. Babelon [134] mentioned that the most frequently represented animal in Carthage steles was the fat-tailed and woolly sheep typical of the Carthage Republic dominating large parts of the western Mediterranean basin, during the 3rd millennium B.P. Carthage was founded by the Canaanite/Phoenician migrated from Canaan in the southern Levant, where pastoral nomadism became the dominant economy, and the fat-tailed sheep became the dominate sheep variety as a response to climate change [126,135]. Unfortunately, the prehistoric written sources of Carthage, which detailed the fat-tailed sheep breeding, as well as the wool sheep production and ancient wool trade in the occidental southern Mediterranean rim, as described in Egyptian and Roman prehistoric sources, were destroyed during the Punic war [136].

The fat deposition in the tail is considered as an adaptive response of sheep to fluctuant environment conditions and is a valuable energy reserve for the animal during migration and food scarcity [84,137]. This westward fat-tailed migration wave, mainly focused on the southern Mediterranean rim, was highlighted by the divergence time (3500 and 2316 years ago, Table 6) between BAR, the unique fat-tailed western Mediterranean breed, and the breeds originating from South Europe as NTH and SS. The fat tail phenotype resulted from the selection of several millennia after domestication for recessive mutations on chromosome 5 and X affecting fat tail size, as even this character would not support a simple single gene control [84]. In fact, during our sampling, we revealed that the population (CRO), resulting from the cross of the fat–tailed BAR rams with thin-tailed QFO ewes, exhibited an intermediate tail. However, the presence of a fat-tailed gene pool in the thin-tailed QFO’s genome and the presence of the thin tailed gene pool in the fat-tailed BAR’s genome was illustrated in the structure analysis, revealing the miscegenation of the thin-tailed sheep of the first migration phase with the fat-tailed sheep of the second migration phase. Moreover, the presence of the fat tail gene pool in the SS and NTH thin-tailed breeds with a European origin, as illustrated by the present structure analysis, as well as the presence of the fat-tailed haplotype in European thin-tailed breeds [138], in addition to the presence of the fat tail cluster in the actual Mediterranean thin-tailed breeds [21,33], further evidenced the miscegenation of the two successive migration phase and the dominance of the thin-tailed phenotype [138].

The second migration phase of these evolved woolly and/or fat-tailed sheep, following the same westward direction from the Middle East, would took a sea way and presented a slower and narrower expansion compared to the first migration phase involving both Neolithic human populations and their livestock, who settled and established their coastal farming enclaves around the Mediterranean Basin [91]. In fact, the second phase of migration took a subsequent seafaring colonization aspect and/or trade exchange between the first states and established civilizations in the Mediterranean basin, which formed an implicit obstacle for a free and vast expansion in time and space [81,91,125].

#### 3.3.3. Third Migration Phase

The third migration phase was highlighted in the present study by the most recent divergence time between the analyzed breeds occurring in the common area between 2000 to 900 years ago (Table 6). This phase was presented by two different aspects:

1. The westward migration wave aspect, which corresponded to the second migration wave reintroducing fat-tailed sheep in the western Mediterranean basin, caused the final divergence between the fat-tailed BAR and the thin-tailed QFO 1440 years ago (Table 6). This divergence time is contemporary to the Islamic and Arab conquest started in the 7th century [139], about 1300 years ago, which concentrated for five centuries mostly in Tunisia, named Ifrikiya in this periode, the capital of the North African and Maghrebian part of the Islamic period [85]. This recent fat-tailed migration wave explained the regeneration of the fat-tailed phenotype only in the southern Mediterranean rim, and its concentration in Eastern North Africa (Egypt, Libya and Tunisia), despite its precedented presence in the western North African thin-tailed sheep genome (Algerian and Moroccan) [25,140], and in the southern European thin-tailed sheep genome, as revealed in the recent genetic structure investigation of Mediterranean breeds [21,33]. These findings justify the dominance of the thin-tailed character [138];
2. The local western Mediterranean gene flow aspect between already installed and more or less evolved and selected woolly Mediterranean breeds. This west Mediterranean breeds’ gene flow finalized the genetic makeup of the present day northern and southern Mediterranean shores’ sheep breeds. This intensive gene flow is strongly related to the wide exchange and trades of finer woolly sheep starting in the Roman period and continues into the medieval period under the merinization process [70,123]. The highest gene flow value revealed between the Algerian-originated QFO breed and Italian APP seems to be an earlier evidence of this process. In fact, the participation of North African sheep in shaping the merino genome was mentioned by Ciani et al. [70]. Unfortunately, the present study did not include merino breeds to highlight the genetic relationship between North African and merino or merino-derived breeds. However, in the clustering analysis result elaborated by Ben Jemaa et al. [33], we revealed the shared genome between thin-tailed Tunisian breeds and merino breeds, with the only difference in the clusters’ proportions. This last study highlighted the strong gen flow and the past introgression, interpreted as unexpected, between the western Mediterranean breeds.

**Figure 7:**
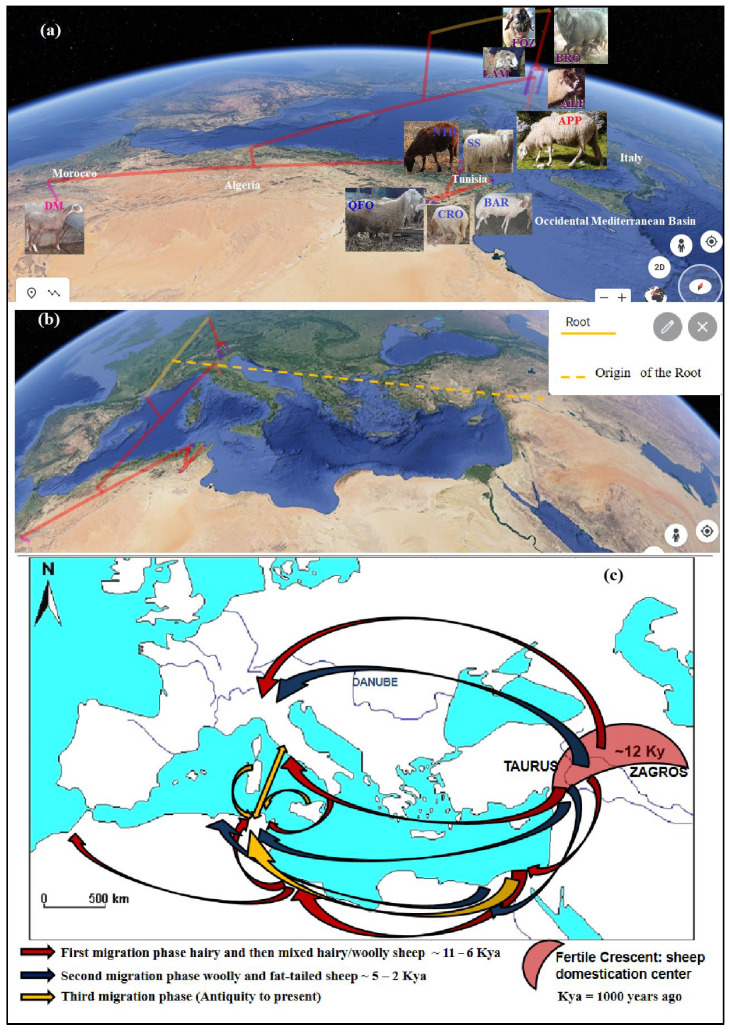
Westward Mediterranean sheep migration phases based on the present microsatellite analysis: (**a**) Mapped (δµ)^2^ phylogenetic tree of the 11 analyzed breeds using GPT method; (**b**) Mapped (δµ)^2^ phylogenetic tree with the assumed root origin using GPT method; (**c**) Mediterranean sheep expansion phases via sea and land routes dating from the Early Neolithic to the present.

In light of these high congruencies between our genetic results and the zooarcheological and historical findings, it is worth speculating that three phases of westward sheep migration, dating from the early Neolithic to the medieval period, shaped the genome of both Mediterranean coasts’ sheep. This tri-phase westward migration was illustrated in Figure 7. The evolutionary and the phylogeographical output of this analysis is clearly visualized in the mapped (δµ)^2^ phylogenetic tree of the 11 analyzed sheep breeds in the occidental Mediterranean basin using the GPT method [61] (Figure 7a), and the assumed origin of their common ancestor (origin of the tree root, Figure 7b). The assumed migration phases scenario is presented in Figure 7c. In fact, the miscegenation of the first phase of the hairy ancestral gene pool, the second phase of the woolly sheep—with firstly coarser and later with finer fleece—and the fat-tailed sheep gene pool, and finally the recent gene flow within this western Mediterranean region, would have drawn the actual Mediterranean sheep’s genetic makeup and participated in the final mosaic genome of the occidental Mediterranean sheep. This three phase introduction assumption is strongly supported by the recent paternal [16,138], maternal [16,32] and genome-wide SNP analysis [21,33].

## 4. Conclusions

The aim of the present study is to investigate the expansion of the western Mediterranean sheep by analyzing native sheep breeds that did not undergo the effect of the strong modern human selection practiced in the last two centuries. A molecular characterization, using microsatellite markers, was successfully used in highlighting the phylogeographic patterns and the divergence times, starting from the early Neolithic to the medieval period, of the westward sheep migration from the domestication center to North Africa and South Europe. The genetic results, strongly supported by systematic classification, zooarcheological, historical and climatic data, contributed to the reconstruction of the history of the westward domestication center sheep migration by the assumption of the three phases’ migration scenario. We highlighted that the current analyzed western Mediterranean breeds were the result of the miscegenation of three migration phases. The first phase, introducing the first ancestral hairy sheep, would have been miscegenated with the second phase of migration sheep. This last more evolved sheep diverged in the Fertile Crescent, after a long selection which looked for desired secondary products and adaptability, leading to the woolly and the fat-tailed sheep that migrated westward. The third migration phase corresponded, in addition to the last introduction of the fat-tailed sheep in the Islamic conquest, to the recent exchange of installed and locally evolved occidental Mediterranean breeds. The miscegenation of these three migration phases shaped the mosaic genome of the westward Mediterranean breeds. Our results perfectly reflected the Neolithic, prehistoric and historic sheep migrations. They contribute to a better visualization of the domestic sheep’s genetic background, since its domestication, as well as the effort that the Neolithic and prehistoric sheep breeders have provided, leads to a productive and well adapted sheep. These sheep, which survived and resisted the extreme cooling and drying climatic events, the fire activity and the desertification that happened during the last postglacial period, were the origin of the native sheep breeds. It is worth emphasizing that these native breeds, with their precious genetic reservoir, deserve to be preserved from the dilution and the intensive selection pressure of productive traits highly practiced during the last two centuries, under the modernization and the production standardization process of breeding.

## Figures and Tables

**Table 1:** Description of morphological traits of the sampled sheep breeds: Maghrebian breeds: Barbarine (BAR), Western Thin-Tailed breed (QFO), crossbred BAR × QFO (CRO), D’man (DM), Sicilo–Sarde (SS), Noire de Thibar (NTH); Venetian breeds: Alpagota (ALP), Brogna (BRO), Foza (FOZ) and Lamon (LAM); Central Italian breed: Appenninica (APP).

Region	Breed	Tail	Horn	Fleece/Coat Color	Photos *
Maghrebian	BAR	Fat tail	Presence in ( 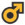 ) rare in ( 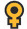 )	Woolly (white or brown fleece)/black or red head and legs or mixed (white and black or red legs, muzzle and eye area)	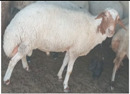
	QFO	Thin tail	Frequent ( 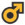 ) low frequency ( 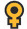 )	Woolly (white fleece)/white coat	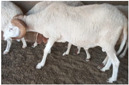
	CRO	Intermediate tail	-	BAR-like fleece and coat	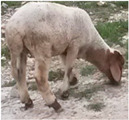
	NTH	Thin tail	Hornless in both sex	Woolly (dark fleece)/dark coat	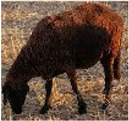
	SS	Thin tail	Presence in both sex	Woolly (white fleece)/white coat	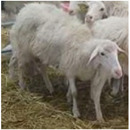
	DM	Long and thin tail	Hornless in both sex	Mixed hairy and woolly with natural molting and presence of hairy man in rams/colored and spotted coat	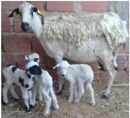
Italian	ALP	Long and thin tail	Hornless in both sex	Woolly (white fleece)/brown-spotted coat	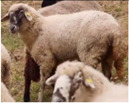
	BRO	Long and thin tail	Rarely present in ( 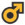 )	Woolly (white fleece)/red-spotted coat	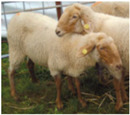
	FOZ	Thin tail	Hornless in both sex	Woolly (white fleece)/black-spotted coat	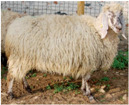
	LAM	Long and thin tail	Hornless in both sex	Woolly (white fleece)/dark brown Spotted coat	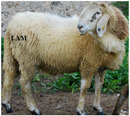
	APP	Long and thin tail	Hornless in both sex	Woolly (white fleece)/white coat	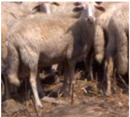

**Table 2:** Characteristics of the microsatellite loci used in the phylogeographic analysis of the occidental Mediterranean sheep breeds.

Locus	Size	Chr	TNA	AR	PIC
Inra023	195–221	1	17	10.25	0.87
Inra063	168–208	14	28	10.40	0.82
OarCP49	71–137	17	31	13.34	0.85
OarFCB304	145–201	19	24	8.96	0.76
OarFCB20	85–121	2	19	10.26	0.84
MAF65	113–139	15	21	7.75	0.75
ILST087	134–184	6	26	13.17	0.88
OarAE119	145–185	19	20	9.51	0.82
MCM527	164–190	5	17	7.89	0.78
MAF214	182–262	16	38	6.96	0.61
OarAE129	135–165	5	16	5.26	0.63
OarCP34	93–117	3	18	6.62	0.77
OarAE54	120–152	25	19	10.44	0.82
TGLA	125–163	12	16	9.67	0.84
URB	159–211	13	24	9.93	0.86
CSRD	208–262	14	28	9.74	0.84
HSC	260–296	20	21	9.33	0.85
Average	-	-	22.53	9.38	0.80
SD	-	-	6.07	2.09	0.08

Chr, chromosome; TNA, number of alleles; AR, allelic richness; PIC, polymorphic informative component; SD, Standard Deviation.

**Table 3:** F_ST_ distances (*p* < 0.001) between the Mediterranean analyzed breeds. Venetian breeds: Alpagota (ALP), Brogna (BRO), Foza (FOZ) and Lamon (LAM); Central Italian breed: Appenninica (APP); Maghrebian breeds: Barbarine (BAR), Western Thin-Tailed breed (QFO), crossbred BAR × QFO (CRO), D’man (DM), Sicilo–Sarde (SS) and Noire de Thibar (NTH).

F_ST_	LAM	FOZ	ALP	BRO	DM	CRO	NTH	QFO	SS	BAR	APP
LAM	-	0.042	0.038	0.039	0.102	0.118	0.114	0.105	0.118	0.118	0.126
FOZ		-	0.053	0.041	0.098	0.110	0.098	0.096	0.111	0.110	0.116
ALP			-	0.040	0.114	0.130	0.126	0.124	0.132	0.137	0.147
BRO				-	0.105	0.114	0.113	0.106	0.122	0.116	0.128
DM					-	0.029	0.034	0.021	0.043	0.032	0.073
CRO						-	0.018	0.006 *	0.023	0.008 *	0.070
NTH							-	0.014	0.020	0.024	0.069
QFO								-	0.018	0.005 *	0.052
SS									-	0.028	0.057
BAR										-	0.067
APP											-

*: nonsignificant.

**Table 4:** Pairwise molecular co-ancestry indexes (*f_ij_*), below the diagonal and effective migrant per generation (Nm), above diagonal. Venetian breeds: Alpagota (ALP), Brogna (BRO), Foza (FOZ) and Lamon (LAM); Central Italian breed: Appenninica (APP); Maghrebian breeds: Barbarine (BAR), Western thin-tailed breed (QFO), crossbred BAR × QFO (CRO), D’man (DM), Sicilo–Sarde (SS), Noire de Thibar (NTH).

*f_ij_*/Nm	DM	CRO	NTH	QFO	SS	BAR	APP	LAM	FOZ	ALP	BRO
DM	-	8.63	7.12	11.23	5.60	7.33	3.20	2.03	2.27	1.80	1.74
CRO	0.17	-	13.89	50.65	10.87	30.54	3.33	1.80	2.08	1.49	1.66
NTH	0.16	0.18	-	18.13	11.80	10.24	3.39	1.78	2.23	1.52	1.64
QFO	0.16	0.18	0.17	-	13.59	43.91	4.55	2.02	2.36	1.60	1.87
SS	0.15	0.17	0.17	0.16	-	8.64	4.06	1.76	1.97	1.45	1.53
BAR	0.16	0.19	0.17	0.18	0.17	-	3.44	1.70	2.02	1.43	1.63
APP	0.15	0.17	0.16	0.17	0.17	0.17	-	1.60	1.85	1.25	1.37
LAM	0.10	0.10	0.09	0.10	0.09	0.09	0.12	-	5.27	3.98	3.24
FOZ	0.10	0.10	0.10	0.10	0.09	0.09	0.11	0.17	-	3.73	4.12
ALP	0.12	0.11	0.10	0.10	0.09	0.09	0.11	0.20	0.18	-	3.06
BRO	0.10	0.10	0.10	0.10	0.09	0.10	0.11	0.18	0.17	0.21	-

**Table 5:** Pairwise genetic distances: (δµ)^2^ below the diagonal and Ds above diagonal. Venetian breeds: Alpagota (ALP), Brogna (BRO), Foza (FOZ) and Lamon (LAM); Central Italian breed: Appenninica (APP); Maghrebian breeds: Barbarine (BAR), Western thin-tailed breed (QFO), crossbred BAR × QFO (CRO), D’man (DM), Sicilo–Sarde (SS) and Noire de Thibar (NTH).

(δµ)^2^/D_S_	LAM	FOZ	ALP	BRO	DM	CRO	NTH	QFO	SS	BAR	APP
LAM	-	0.186	0.142	0.162	0.486	0.541	0.532	0.509	0.564	0.555	0.534
FOZ	1.652	-	0.205	0.169	0.475	0.510	0.461	0.474	0.536	0.521	0.492
ALP	1.757	0.932	-	0.147	0.482	0.538	0.528	0.538	0.563	0.585	0.571
BRO	1.753	1.116	1.679	-	0.478	0.499	0.501	0.492	0.558	0.521	0.523
DM	5.949	6.402	5.635	6.442	-	0.132	0.156	0.108	0.201	0.146	0.298
CRO	2.752	2.841	3.102	3.391	2.470	-	0.079	0.030	0.104	0.037	0.275
NTH	3.069	3.141	3.801	4.471	3.578	0.458	-	0.065	0.090	0.105	0.273
QFO	2.503	2.829	3.146	3.633	3.508	0.692	0.540	-	0.089	0.025	0.213
SS	2.118	2.266	3.074	3.208	4.055	0.925	0.717	0.998	-	0.125	0.230
BAR	3.165	3.023	3.325	3.258	3.378	1.150	1.390	0.864	2.149	-	0.267
APP	3.220	3.852	5.869	2.954	6.609	3.340	3.756	3.899	2.366	4.166	-

**Table 6:** Divergence time in years ago between breeds calculated from (δµ)^2^ distances. Venetian breeds: Alpagota (ALP), Brogna (BRO), Foza (FOZ) and Lamon (LAM); Central Italian breed: Appenninica (APP); Maghrebian breeds: Barbarine (BAR), Western thin-tailed breed (QFO), crossbred BAR × QFO (CRO), D’man (DM), Sicilo–Sarde (SS) and Noire de Thibar (NTH).

Y. Ago	LAM	FOZ	ALP	BRO	DM	CRO	NTH	QFO	SS	BAR	APP
LAM											
FOZ	2753.3										
ALP	2928.3	1553.3									
BRO	2921.7	1860.0	2798.8								
DM	9915.0	10,670.0	9392.0	10,736.3							
CRO	4586.7	4735.0	5169.2	5652.4	4117.4						
NTH	5115.0	5235.0	6334.2	7451.7	5963.3	763.8					
QFO	4171.7	4715.0	5243.7	6055.0	5847.4	1154.0	900.8				
SS	3530.0	3776.7	5123.7	5346.4	6758.7	1541.2	1195.6	1663.6			
BAR	5275.0	5038.3	5542.5	5430.6	5629.6	1916.6	2316.3	1440.8	3582.3		
APP	5366.7	6420.0	9781.8	4922.8	11,015.0	5566.3	6260.8	6498.1	3943.4	6943.2	

## Data Availability

Data presented in this study are available within the article.
